# Hemp Genome Editing—Challenges and Opportunities

**DOI:** 10.3389/fgeed.2022.823486

**Published:** 2022-02-02

**Authors:** Donal Shiels, Barbara Doyle Prestwich, Okjae Koo, Chidananda Nagamangala Kanchiswamy, Roisin O'Halloran, Raghuram Badmi

**Affiliations:** ^1^ School of Biological Earth and Environmental Sciences, Environmental Research Institute, University College Cork, Cork, Ireland; ^2^ Plantedit Pvt Ltd, Cork, Ireland

**Keywords:** HEMP, non-transgenic, genome editing, cannabinoids, tissue culture, next generation technologies, artificial intelligence for crop improvement

## Abstract

Hemp (*Cannabis sativa* L.) is a multipurpose crop with many important uses including medicine, fibre, food and biocomposites. This plant is currently gaining prominence and acceptance for its valuable applications. Hemp is grown as a cash crop for its novel cannabinoids which are estimated to be a multibillion-dollar downstream market. Hemp cultivation can play a major role in carbon sequestration with good CO_2_ to biomass conversion in low input systems and can also improve soil health and promote phytoremediation. The recent advent of genome editing tools to produce non-transgenic genome-edited crops with no trace of foreign genetic material has the potential to overcome regulatory hurdles faced by genetically modified crops. The use of Artificial Intelligence - mediated trait discovery platforms are revolutionizing the agricultural industry to produce desirable crops with unprecedented accuracy and speed. However, genome editing tools to improve the beneficial properties of hemp have not yet been deployed. Recent availability of high-quality *Cannabis* genome sequences from several strains (cannabidiol and tetrahydrocannabinol balanced and CBD/THC rich strains) have paved the way for improving the production of valuable bioactive molecules for the welfare of humankind and the environment. In this context, the article focuses on exploiting advanced genome editing tools to produce non-transgenic hemp to improve the most industrially desirable traits. The challenges, opportunities and interdisciplinary approaches that can be adopted from existing technologies in other plant species are highlighted.

## Introduction

Cultivation of hemp (*Cannabis sativa*) has increased globally in recent years and is a profitable enterprise that generates a range of useful products such as bioactive cannabinoids, seed, seed oil, fibre, textiles, construction materials and biocomposites. Archaeological evidence from Western China dating from 500 BCE suggests *Cannabis* was used for ceremonial purposes by ancient Chinese cultures during burial ceremonies (Ren et al., 2019). The medicinal properties of cannabinoids are extensively documented and renewed interest in these compounds in recent decades has driven growth in the health product and medical markets. The classification of *Cannabis* is typically determined by plant chemistry. In Europe, hemp was defined as *Cannabis sativa* plants containing less than 0.2% of the intoxicating cannabinoid Δ9-tetrahydrocannabinol (THC), but recent changes to laws and the adoption of the new Common Agricultural Policy have increased this to less than 0.3%. This figure is less than 0.3% in North America and Asia (Russo, 2017; Hammami et al., 2021). Drug-type *Cannabis* plants are grown for their high levels of the intoxicating THC and are commonly referred to as marijuana. *Cannabis* is a reservoir for a range of valuable secondary metabolites including cannabinoids and terpenes. Cannabinoids that have documented medical properties include cannabidiol (CBD), cannabigerol (CBG), cannabinol (CBN), cannabichromene (CBC), cannabidiolic acid (CBDA), cannabidivarin (CBDV), cannabicyclol (CBL), cannabivarin (CBV), cannabichromevarin (CBCV) and cannabielsoin (CBE). Cannabinoids accumulate in the secretory cavity of the hair-like glandular trichomes which are found in greatest abundance on the female inflorescences (Livingston et al., 2020). CBD is one of the most prominent non-intoxicating cannabinoids that has potential in treatment of various medical conditions including epilepsy, chronic pain, autism and post-traumatic stress disorder. Currently, medical *Cannabis* is legal in more than 50 countries including China, Australia, Germany, Israel, Canada and most of the U.S. The medical *Cannabis* market is rapidly growing from $3.5 billion at retail prices in 2019 to an estimated $20.2 billion during 2020–2025 (Aliekperova et al., 2020). Hemp is one of the earliest documented fibre crops used by humans with claims of domestication as early as 12,000 years ago (Ren et al., 2021). Hemp fibre is a strong, durable material with good insulative properties. It is used to make clothing, textiles, building materials and polymers. Hemp-based bioplastics have shown potential and could be superior in some respects to traditional polymers. They also offer a more sustainable, greener alternative to petroleum-based plastics (Fike, 2016). Materials such as Hempcrete^®^ offer a means of carbon-negative building methods which can reduce net greenhouse gas emissions (Ip and Miller, 2012). Industrial hemp is an excellent carbon sink. Finnan and Styles (2013) found that hemp is comparable to the energy crops miscanthus and short-rotation coppice willow in net greenhouse gas emission abatement, and superior to sugar beet and oil seed rape. A comparative study carried out in Sweden demonstrated how hemp had similar biomass energy yield to maize and sugar beet (Prade et al., 2011). Hemp also has potential as a break crop between the planting of two food crop cycles and could play an important role in sustainable farming. This strategy can reduce soil pathogens, improve soil structure, and enrich soils if crop residues are ploughed in. Studies have shown how food crops such as wheat (Gorchs et al., 2017) and soybean (Liu et al., 2012) benefit from increased yield after hemp breaks crops over continuous systems. This accounted for yield increases of 37–48% in wheat monocultures and 9.1–10.8% in soybean monocultures. Hemp has documented nematicidal properties also and some of these yield gains can be attributed to suppression of these parasites (Adesina et al., 2020). Demand for hemp seed, oil and press-cake (remains of seed once pressed for oil) has contributed to the increased cultivation of hemp in the US (Adesina et al., 2020). As a food source hemp-derived protein has high nutritional value and excellent digestibility. The seed contains all the essential amino acids required by humans. There are also reported health benefits from consumption of hemp-derived protein including decreasing hypersensitivity and cholesterol (Shen et al., 2021). Overall, the cultivation of hemp has clear benefits and there is a growing market for hemp-derived products. Maximising the potential of this plant calls for more high-performing cultivars. Developing new cultivars through traditional breeding can often take a lot of time and labour. However, molecular breeding strategies such as marker assisted selection have refined the process of breeding, but these strategies are not well developed in this species. The recent development and adoption of genome editing technologies such as CRISPR (clustered regularly interspaced short palindromic repeats**)** offers a means to improve hemp varieties in a more precise and less time-consuming way. Many hemp varieties don’t self-pollinate, and this prevents using this strategy to obtain homozygous plants. Gene-editing technology allows breeders to modify genes on both alleles to achieve homozygous lines in one generation (Deguchi et al., 2020). Potential targets for gene-editing in hemp include genes controlling cannabinoid production and accumulation, fibre deposition, disease susceptibility and seed oil quality. This review discusses the opportunities for improving hemp with gene editing technology, and the potential challenges and opportunities in adopting these technologies.

## Targets for Hemp Crop Improvement

The many different uses of *Cannabis* motivate the development of high performing cultivars with improved cannabinoid production, fibre accumulation, disease resistance and food quality. The growing demand for cannabinoids means there is an opportunity to develop high-yielding cultivars using novel methods. However, more research is needed to understand potential trade-offs when applying this technology. A knockout of the THC acid synthase gene via genome editing is a way to derive THC-free, high-CBD plants which would have huge value in countries with strict laws on THC levels. A patent filed by Canopy Growth Corporation details overexpression of genes regulating trichome development (e.g. GLABROUS INFLORESCENCE STEMS (GIS)) produced trichomes in greater density and abundance, and had a ten-fold increase in THC production over unmodified plants (Roscow, 2019). Using non-transgenic genome editing technology, the target gene(s) can be overexpressed by editing the respective regulatory (enhancer/promoter) elements upstream of a gene. Genome editing has been successfully applied to other important medicinal plant species such as *Dendrobium officinale*, *Papaver somniferum*, *Dioscorea zingiberensis* and *Salvia miltiorrhiza* (Alagoz et al., 2016; Kui et al., 2017; Feng et al., 2018; Zhou et al., 2018) and there is huge scope to modulate metabolite production via CRISPR/Cas9. Fibre quality of cultivated hemp plants can be improved by upregulating the expression of genes involved in the formation of bast fibres (phloem fibres). Several well-known transcription factors including NST1, MYB46 and WILM1 control secondary cell wall deposition and bast fibre development in hemp hypocotyls. The genes SND2, VND1 and NST1 are master regulators of secondary cell wall development (Behr et al., 2016). Hemp is susceptible to a range of diseases that can lead to loss in yield and decrease the overall value of the crop. Common pathogens of hemp include fungi, oomycetes, viruses, nematodes, and bacteria. Genome editing technologies offer a way to generate disease resistant varieties with greater precision and in a faster time frame than traditional breeding methods. Targeting resistance (R) and susceptibility (S) genes are one way to increase a plant’s resistance. A recent study has identified a powdery mildew resistance (R) gene in a *Cannabis sativa* cultivar, designated PM1, that confers resistance to the pathogen *Golovinomyces ambrosiae* (Mihalyov and Garfinkel, 2021). Improving food quality of seed and seed oil is also possible. Targeting *FAD2* genes which are involved in converting oleic acid to linoleic acid and linolenic acid offer a reliable target to upregulate oleic acid production. Mutagenesis studies on the hemp cultivar Finola have shown increased oil quality (high oleic content) and shelf life through targeted mutations of fatty desaturase genes *CsFAD2* and *CsFAD3* (Bielecka et al., 2014). Genome editing of *Brassica napus* fatty acid desaturase gene 2 (*FAD2*) using CRISPR/Cas9 has been successfully demonstrated, producing high oleic acid content seed. Back-crossing of the progeny of one transformed line showed the mutation was inheritable and no transgenic DNA was inherited (Okuzaki et al., 2018).

## Opportunities and Challenges for Hemp Improvement Using Next Generation Tools

### DNA-free/footprint-free Genome Editing

**FIGURE 1:**
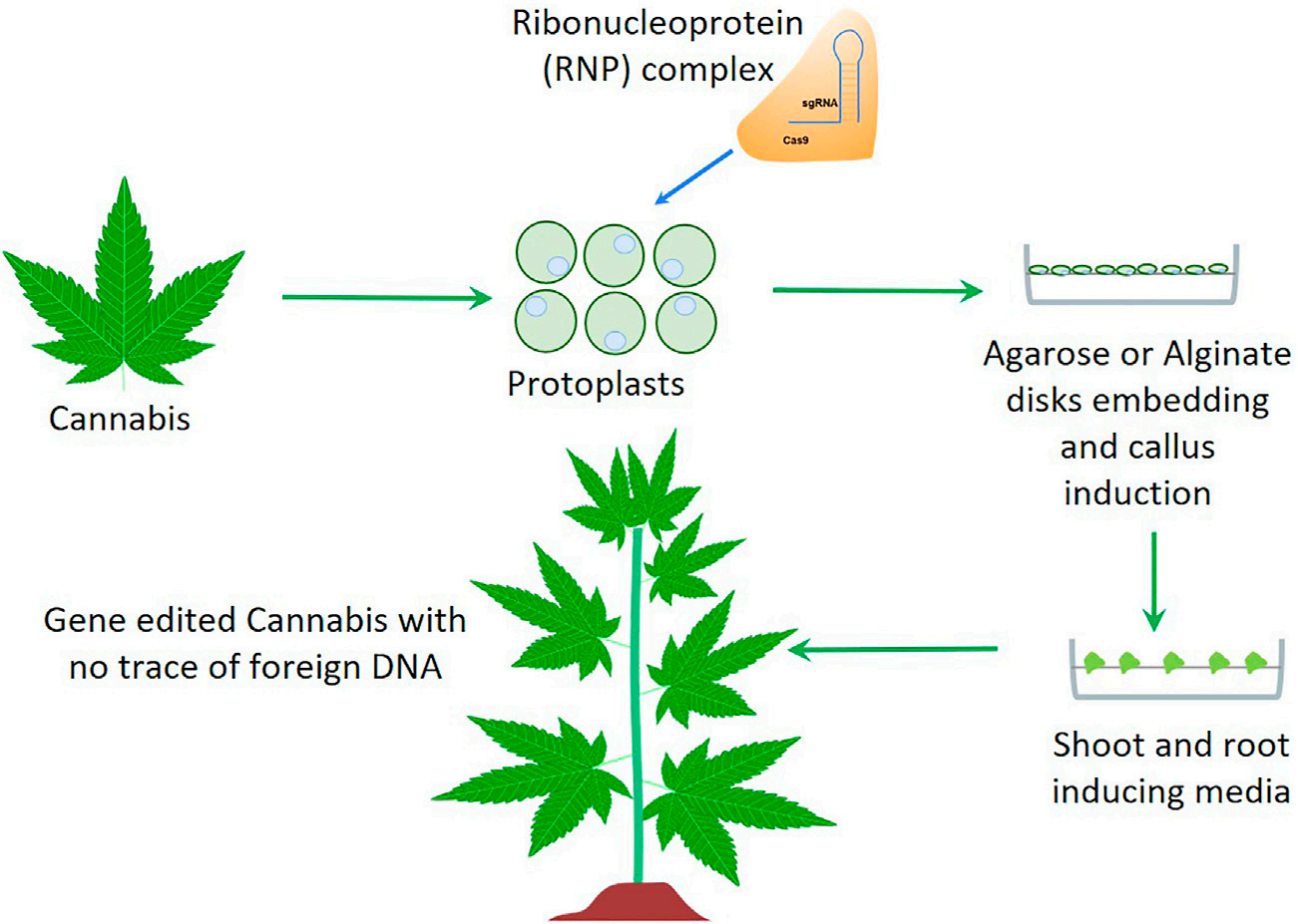
A schematic overview of non-transgenic genome editing technology applicable for *Cannabis* improvement. *Cannabis* protoplasts isolated from *in-vitro* grown plants are transfected with a mixture of Cas9 and sgRNA followed by agarose or alginate embedding and plant regeneration.

Genome Editing by CRISPR/Cas is revolutionizing plant biology and agriculture in developing improved crops with novel traits. CRISPR/Cas technology allows for sequence specific editing of the target genome, thereby allowing for precise control over gene modifications and associated traits, in a low cost and straightforward manner. This level of control over DNA sequence change is unprecedented. It is a vast improvement over previous genome modification tools and opens new doors for exciting developments in the fields of medicine and agriculture. Agrobacterium-mediated CRISPR transformation is being widely used for targeted crop improvement to develop gene knockouts, knock-ins, transcriptional regulation, and epigenetic changes in the genome to achieve novel traits. However, agrobacterium-mediated transformation may pose a bottleneck for regulatory approval because of the introduction of external plasmid DNA into the plant genome. The newest next-generation genome editing technology encompasses modifying/editing the DNA bases by direct delivery of CRISPR/Cas ribonucleoprotein (RNP) complexes into plant tissue, such as protoplasts, embryos or *in-vitro* grown calli (Woo et al., 2015; Malnoy et al., 2016; Osakabe et al., 2018). The transformed plant tissue is grown in a suitable media to regenerate entire plants followed by screening for the genome edited plant lines (Figure 1). This approach eliminates the opportunity for plasmid encoded DNA elements to integrate into the plant genome, thereby mimicking natural mutations. In addition to introducing mutations and deleting entire fragments of DNA elements, CRISPR/Cas technology is also being used to introduce a specific DNA fragment to a precise location in the genome. A specific donor DNA is included together with Cas9 and sgRNA which spans the flanking regions of the target site with the donor DNA element in between. The presence of this single-stranded DNA triggers the Homology Directed Repair (HDR) mechanism wherein the donor DNA is used as a template by the DNA repair machinery to repair the cut target site, and consequently the target DNA sequence gets introduced to the target genome (Chen et al., 2019). This new generation of precision methods has several applications in hemp breeding such as gene knockout/knock-in, base editing, gene- and genome-wide screening, modifying gene regulation, and developing virus resistant plants, as demonstrated in different recalcitrant species such as wheat, maize and grape. These strategies have been clearly detailed in the review article by Chen et al. (2019) by providing specific examples.

### Interdisciplinary Approaches for Hemp Biology

The availability of cannabis genome sequences (Braich et al., 2020; Gao et al., 2020) and growing number of RNA-sequencing datasets (Massimino., 2017; Braich et al., 2019; Braich et al., 2019, 2019; Zager et al., 2019; Livingston et al., 2020; Livingston et al., 2020, 2020) allows for the use of big data analysis methods for greater understanding of *Cannabis* biology beyond the expression levels of genes. A systems-biology approach uses information-rich complex datasets to provide meaningful results by extrapolating the relationship between individual biomolecules. Biomolecules (e.g. genes, transcription factors, metabolites, promoters) are represented as nodes and the connections between them as edges in this in-silico molecular network. Two molecules (nodes) connected by an edge would mean a possible interaction in terms of physical interaction, biosynthesis, regulation and/or co-expression between them. Once the networks are developed, the dynamics of the interactions can be studied with a focus on the hubs that can be central to a biological function of interest (Breitling, 2010). This integrated approach is quite useful to make sense of the vast amounts of datasets produced by holistic studies and will provide a combined biological insight (emergent behaviour) that isolated experiments simply cannot. New genes participating in defense response pathways are predicted and validated using a systems biology approach in *Arabidopsis thaliana* (Windram et al., 2012). This emphasizes the potential of interdisciplinarity in biological research. Furthermore, combining the metabolomics datasets to develop network models using machine learning has been successful in predicting metabolic pathways in tomato (Toubiana et al., 2019). Applying these established methods in hemp will speed-up the understanding of molecular processes and metabolite accumulations in the context of improving desirable traits in hemp such as higher CBD production.

Another application of computational methods is the use of Artificial Intelligence (AI) to identify single nucleotide polymorphisms (SNPs) associated with important agricultural traits in Genome Wide Association Studies (GWAS). By using the available genomic sequences from different varieties of a certain crop species, these deep learning-based prediction methods can identify SNPs associated with the trait of interest. The machine learning algorithms are first trained with a combination of data including genotypic, phenotypic, agronomic practices and environmental data before it is used on a test dataset for predicting SNPs (Wang et al., 2020; Mieth et al., 2021). This is just one of the applications of AI and deep learning to accelerate knowledge discovery. The review article by Wang et al. (2020) provides a good overview of its various applications in plant research and agriculture. GWAS studies have been carried out on hemp with respect to fibre quality (Petit et al., 2020a) and flowering time and sex determination (Petit et al., 2020b). Hesami et al. (2021a) applied machine learning algorithms *in silico* to predict off-target gRNA activity in modifying centromeric histone H3 (CENH3) genes in *Cannabis*. Of the three machine learning algorithms used, the Random Forest (RF) had the highest precision. These predictive models offer a powerful tool in designing effective genome-editing protocols in *Cannabis*. Interdisciplinary approaches will accelerate the knowledge-discovery and will be valuable to understand cannabinoid biology and genetics, given imperfect genome sequence and annotations, recalcitrance for transformation and the lack of standard protocols/procedures for Cannabis.

### Micropropagation and Plant Regeneration

Micropropagation of *Cannabis* tissues in a disease-free aseptic environment is an important step towards a successful transformation protocol. Some varieties of *Cannabis* are recalcitrant to *in vitro* culture and transformation. An optimal strategy may be to transform more amenable varieties and backcross these into elite lines, which is still time and labor intensive. Adhikary et al. (2021) mentions that the *Cannabis* industries have been developing tissue culture and micropropagation techniques over the last 2 decades and are held as a trade secret to preserve competitive advantage with other commercial entities. Optimizing micropropagation protocols for non-meristematic tissues is important for genome editing applications. Factors including plant growth regulators (PGRs), type of light, carbohydrate sources, additives, temperature and genotype influence micropropagation success (Hesami et al., 2021b). Zhang et al. (2021) found that *Cannabis* embryo hypocotyls of immature grains collected 15 days after anthesis exhibited the greatest regeneration rate and were also more amenable to agrobacterium transformation. The authors used G41sg vector to deliver sgRNA targeting phytoene desaturase gene (CsPDS1) generating albino plants. This is the first published report of successful gene editing in *Cannabis sativa*, which paves the way for further developments in non-transgenic genome editing technology. Regenerating transfected protoplast cells into complete plants is also challenging. The first report of DNA-free (or non-transgenic) genome editing described the successful regeneration of genome edited lettuce protoplasts into complete plants (Woo et al., 2015). The authors incubated preassembled complexes of purified Cas9 protein and guide RNA with plant protoplasts in the presence of polyethylene glycol (PEG), a standard and widely used transfection method. Interestingly, RNA-guided mutations were detected as early as 24 h, suggesting the quick Cas9 activity even before the cell cycle was completed. The transfected protoplasts were mixed with a 1:1 solution of 50% B5 medium and 2.4% agarose to make agarose embeddings plated on 6-well plates (Woo et al., 2015). For regenerating plants from genome edited grapevine protoplasts the authors embedded the protoplasts in alginate disks and stimulated the formation of mini-calli in NN-based cultivation medium (Nitsch and Nitsch, 1969) optimized for regeneration (Scintilla et al., 2021). Beard et al. (2021) demonstrated PEG mediated transient transformation of *Cannabis* sativa protoplasts with a p35S:GFP expression cassette and achieved a transformation efficiency of up to 31%, thus demonstrating the viability of protoplast transformation in this species. Table 1 lists the published transformation and regeneration technologies for non-transgenic genome editing in different plants. Effective protoplast culture protocols provide a platform for whole plant regeneration, and a platform to test sgRNAs in optimizing CRISPR protocols.

**TABLE 1:** Protoplast transformation and regeneration technologies in different species applicable for *Cannabis* improvement.

DNA-free GE technology	Crop/Tissue	Method overview	Reference
Transformation and Regeneration	Wheat Immature Embryos	CRISPR/Cas9 is delivered as DNA (plasmid constructs) or RNA (*in vitro* synthesized transcripts) into immature wheat embryos by particle bombardment, transferred onto callusing media from which seedlings are regenerated	Zhang et al. (2016)
Transformation and Regeneration	Maize Immature Embryos	Guide RNA–Cas9 ribonucleoprotein (RNP) complexes are delivered into maize embryo cells, cultured, selected on appropriate antibiotics and the plants regenerated	Svitashev et al. (2016)
Transfection and Regeneration	Grapevine protoplasts	Protoplasts immobilized in alginate disks were stimulated for mini-calli formation followed by embryo formation and plant regeneration	Scintilla et al. (2021)
Transfection in all four and Regeneration only in lettuce	Arabidopsis, tobacco, rice and lettuce protoplasts	PEG mediated transfection of sgRNA-Cas9 RNP complexes into protoplasts and mixed with a 1:1 solution of 0.5× B5 medium and 2.4% agarose to make agarose embeddings, which were cultured onto callus inducing medium and subsequently transferred to shoot inducing and root inducing media	Woo et al. (2015)
Transfection only	Apple and Grapevine protoplasts	PEG mediated transfection of sgRNA-Cas9 RNP complexes into protoplasts	Malnoy et al. (2016)
Transfection only	*Petunia hybrida* protoplasts	PEG mediated transfection of sgRNA-Cas9 RNP complexes into protoplasts	Subburaj et al. (2016)
Regeneration only	Potato Protoplasts	Protoplasts immobilized in alginate lens are transferred onto callus induction media, and the resulting calli to proliferation media and then to greening media	Moon et al. (2021)
Callus formation	Arabidopsis shoot and root protoplasts	Detailed molecular methods to confirm every stage of protoplast regeneration, special medium designed for Totipotent cell formation, protoplasts immobilized in alginate beds for colony formation	Pasternak et al. (2021)
Regeneration only	Strawberry protoplasts	Isolated protoplasts are embedded in 0.6% agarose and transferred onto regeneration media	Barcelo et al. (2019)

In another approach, researchers used immature embryos from wheat and maize to bombard the mixture of either CRISPR/Cas RNPs or DNA/RNA elements encoding Cas proteins with sgRNAs coated on microparticles (Svitashev et al., 2016; Zhang et al., 2016; Liang et al., 2017, 2018). The embryos were transferred to callusing media, and then to shoot and root regeneration media for complete plant development. This procedure can be applied to develop non-transgenic *Cannabis* plants by using an embryo extraction protocol (Soler et al., 2016). The following biolistic transformation and whole plant regeneration method needs optimization in *Cannabis*.

Co-transformation of developmental regulator genes in combination with the target genes of interest have proven to increase or induce callus formation in recalcitrant varieties of sorghum, maize and wheat (Che et al., 2021; Hoerster et al., 2020; Nalapalli et al., 2021). In *Cannabis*, co-transformation of native homologs of developmental regulators in combinations increased shoot regeneration efficiency up to 1.7-fold with CsGRF3–CsGIF1 chimera and all chimeras containing CsWUS4 (Zhang et al., 2021). WUSCHEL (WUS) is essential for *de novo* establishment of the shoot stem cell niche (Zhang et al., 2017) and co-transfecting WUS into protoplasts could induce the formation of calli and subsequently shoots.

## Discussion

The current legal status of gene-edited crops in the European Union as genetically modified organisms (GMOs) prevents the full deployment of these technologies in *C. sativa*. A move toward social acceptance of gene-edited crops requires a raising of public awareness and a clear distinction between transgenic and non-transgenic plants. Highlighting the use of random mutagenesis by chemical and radiological means for the last century in developing new crop varieties offers utility in advocating the use of targeted mutagenesis technologies. Strictly speaking, DNA-free, gene-edited crops are equivalent to crop varieties derived through random mutagenesis, which include many important food crop species (e.g. bananas, barley). The caveat being that targeted mutagenesis is not random and offers greater control and specificity and reduces the incidence of deleterious mutations and the impact of mutation load (Jung and Till, 2021). The ruling of the Court of Justice of the European Union in 2018 on genome editing groups this new technology with GMOs as outlined in directive 2001/18. The distinction of gene-edited crops as genetically modified organisms (GMOs) within the EU also excludes them from organic certification. This may serve as a hurdle in the public acceptance of these crops as healthy, safe and nutritious. This contentious decision has been challenged by the European Federation of Biotechnology (EFB). They argue that the Site Directed nuclease 1 (SDN1) format of gene editing is fundamentally different from the genetic engineering outlined in directive 2001/18. Safety concerns of introducing foreign DNA and causing off-target mutations are avoided in SDN1 as no foreign DNA is introduced and whole genome sequencing of the transformed organism can investigate any potentially dangerous mutations (Hjort et al., 2021). The recent refinement of CRISPR methods circumvents the issue of introducing transgenes into gene-edited crops, where *Agrobacterium* plasmid DNA is not used, and nucleases are delivered directly into the cells (Ishii, 2018). Even though hemp has large genetic diversity and traditional breeding still offers utility to improve varieties, application of these new plant breeding technologies allows highly specific changes in markedly shorter timeframes. In producing new allelic variation in crop species, CRISPR is the most powerful tool available to breeders, and should be exploited for its full potential.
